# Spatially restricted loading of BRD2 at DNA double-strand breaks protects H4 acetylation domains and promotes DNA repair

**DOI:** 10.1038/s41598-017-13036-5

**Published:** 2017-10-10

**Authors:** Ozge Gursoy-Yuzugullu, Chelsea Carman, Brendan D. Price

**Affiliations:** Department of Radiation Oncology, Dana-Farber Cancer Institute, Harvard Medical School, 450 Brookline Avenue, Boston, MA 02215 USA

## Abstract

The n-terminal tail of histone H4 recruits repair proteins, including 53BP1, to DNA double-strand breaks (DSB) and undergoes dynamic acetylation during DSB repair. However, how H4 acetylation (H4Ac) recruits repair proteins and reorganizes chromatin during DNA repair is unclear. Here, we show that the bromodomain protein BRD2 is recruited to DSBs. This recruitment requires binding of BRD2’s tandem bromodomains to H4Ac, which is generated at DSBs by the Tip60/KAT5 acetyltransferase. Binding of BRD2 to H4Ac protects the underlying acetylated chromatin from attack by histone deacetylases and allows acetylation to spread along the flanking chromatin. However, BRD2 recruitment is spatially restricted to a chromatin domain extending only 2 kb either side of the DSB, and BRD2 does not spread into the chromatin domains flanking the break. Instead, BRD2 facilitates recruitment of a second bromodomain protein, ZMYND8, which spreads along the flanking chromatin, but is excluded from the DSB region. This creates a spatially restricted H4Ac/BRD2 domain which reorganizes chromatin at DSBs, limits binding of the L3MBTL1 repressor and promotes 53BP1 binding, while limiting end-resection of DSBs. BRD2 therefore creates a restricted chromatin environment surrounding DSBs which facilitates DSB repair and which is framed by extensive ZMYND8 domains on the flanking chromatin.

## Introduction

The repair of DNA double-strand breaks (DSBs) requires recruitment of DNA repair proteins to the site of damage and is linked to changes in nucleosome dynamics and histone modification^1,2^. This fundamental reorganization of chromatin is required to promote access of the repair machinery to the DSB and to facilitate repair processes such as end-resection and homology search during homologous recombination. The repair of DSBs therefore requires a tightly coupled partnership between chromatin remodeling complexes and the DNA repair machinery.

DSB repair promotes a rapid, PARP-dependent recruitment of repressive complexes to the chromatin, followed by a coordinated shift to a more open, flexible chromatin conformation^2^. These repressive factors include HDACs and the NuRD remodeling complex^3^, HP1 and the H3K9 methyltransferase SUV39H1^4–6^ and histone demethylases^7^. H2A.Z is also exchanged onto the chromatin at DSBs (by NuA4-Tip60^8,9^), creating H2A.Z-nucleosomes with an enlarged acidic patch. The unacetylated H4 tail then binds to the acidic patch^8,9^, promoting nucleosome interaction and formation of compact chromatin at the DSB^9–11^. This repressive chromatin may restrict local nucleosome motion, limit transcription and erase local epigenetic modifications which may inhibit repair^12^. These temporary repressive structures are dismantled by removal of H2A.Z^9^ and exchange of other histone variants at DSBs^13–15^. This promotes H2A ubiquitination^16,17^ and acetylation of H4 (by KAT5/Tip60^10,18,19^). Together, these histone modifications create an open, acetylated chromatin structure which is required for efficient DSB repair^10,20^.

The H4 tail functions as a central hub for regulating recruitment of DSB repair proteins to the site of damage. 53BP1, which plays a central role in directing repair to either the HR or NHEJ repair pathways^21^, is recruited to DSBs through dual binding to H2A ubiquitinated at lysines 13 and 15 (H2AK13/15)^22,23^ and H4K20me2^24^. H2A is ubiquitinated by RNF168 in response to DNA damage^22,23^, whereas 53BP1 relies mainly on pre-existing H4K20me2. However, 2 proteins which bind to H4K20me2, the repressor L3MBTL1^25^ and the lysine demethylase KDM4A^26^, can block 53BP1 loading at DSBs. Consequently, the VCP/p97 ATPase complex is recruited to DSBs where it actively removes L3MBTL1 and promotes 53BP1 binding^25^. Further, acetylation of H4 at lysine 16 (H4K16Ac) can limit interaction of 53BP1 with H4K20me2 and regulate 53BP1 recruitment to DSBs^27^. The H4 tail, and access to H4K20me2, is therefore critical for facilitating H4 acetylation, the loading of 53BP1 and for the correct processing of the chromatin during DSB repair.

The H4 tail contains multiple lysine residues which are acetylated by NuA4-Tip60 in response to DNA damage^10,18,19,27^. Studies have shown that the acetylated H4 tail provides a binding surface for a number of bromodomain proteins^28,29^. This includes ZMYND8, which recruits the repressive NuRD complex to DSBs and promotes transcriptional silencing during repair^29,30^ and BRD4, which may limit spreading of DNA damage signals along the chromatin^31^. However, how histone acetylation signatures created by DNA damage recruit specific bromodomain proteins to DSBs is currently unknown. Here, we show that the bromodomain protein BRD2 is rapidly recruited to H4Ac at DSBs where it forms a spatially restricted domain extending 2 kb either side of the break. BRD2 therefore defines a novel, spatially restricted chromatin domain which surrounds the DSB and is essential for DSB repair.

## Results

### BRD2 is recruited to H4Ac at DSBs

**Figure 1:**
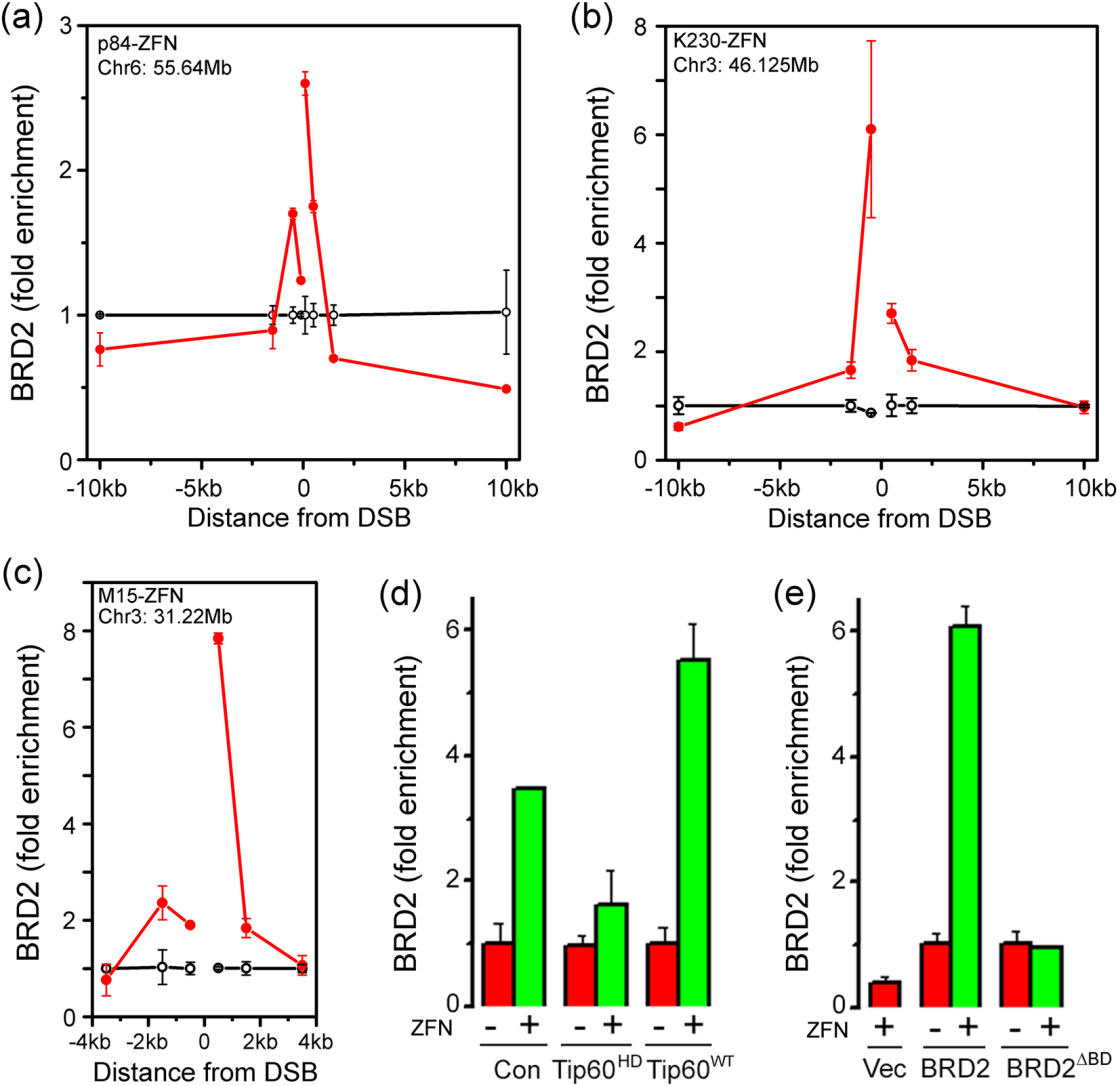
Recruitment of BRD2 to acetylated H4 at DSBs. (**a**–**c**) 293 T cells were transfected with vector (o) and either (**a**) p84-ZFN (); (**b**) M15-ZFN () or (**c**) K230-ZFN (). 18hr later, ChIP was carried out using BRD2 antibody and primers located either side of the DSB. (**d**) 293 T cells expressing vector (Con), HA-Tip60^WT^ or catalytically inactive HA-Tip60^HD^ were transfected with vector (−) or p84-ZFN (+) and processed for ChIP using BRD2 antibody and primers 500 bp to the right of the DSB. **(e)** 293 T cells expressing vector (Vec), YFP-BRD2 or YFP-BRD2^ΔBD^, containing inactivating mutations in both BRD2 bromodomains, were transfected with vector (−) or p84-ZFN (+) and processed for ChIP using BRD2 antibody and primers 500 bp to the right of the DSB. All ChIP data calculated as IP/Input, and expressed as fold enrichment in signal relative to the uncut DNA (n = 3 replicates, ± Standard Deviation).

BRD2 contains 2 tandem bromodomains which bind acetylated H4^32^. To determine if BRD2 was recruited to DSBs, Zinc Finger Nucleases (ZFNs) were used to create DSBs in the non-essential PPP1R12C gene (p84-ZFN)^4,8^ or at 2 intergenic sites at position 46.125 Mb (K230-ZFN) and 31.22 Mb (M15-ZFN) on chromosome 3^33^. BRD2 was monitored by Chromatin Immunoprecipitation (ChIP) and real-time quantitative PCR using a validated BRD2 antibody (Supplementary Figure 1a). BRD2 accumulated at all 3 ZFN DSBs (Fig. 1a–c). However, unlike most DSB repair proteins, BRD2 accumulation was restricted to a region approximately 3–4 kb either side of all 3 DSBs (Fig. 1a–c). BRD2 was also reduced on the chromatin flanking the p84-ZFN DSB (Fig. 1a) and this loss extended at least 50 kb from the DSB (Supplementary Figure 1b). BRD2 is a transcriptional regulator^34^, and p84-ZFN creates a DSB in the transcribed PPP1R12C gene. Loss of BRD2 at the p84-ZFN DSB may reflect removal of pre-existing BRD2 from the PPPIR12C gene after DNA damage. Overall, Fig. 1a–c demonstrates that BRD2 is actively concentrated onto nucleosomes directly adjacent to DSBs.

Because BRD2 binds to H4K8Ac and H4K12Ac^35,36^ and H4 is acetylated by Tip60 after DNA damage^10,18,27^, we examined if BRD2 recruitment required Tip60. Expression of wild type Tip60 enhanced loading of BRD2 at DSBs (Fig. 1d) whereas a catalytically inactive Tip60 (Supplementary Figure 1c) blocked BRD2 recruitment (Fig. 1d). This is consistent with BRD2 loading requiring H4 acetylation by the Tip60 acetyltransferase. Next, the tyrosines in BRD2’s tandem bromodomains which are required for binding to H4Ac were mutated to phenylalanine^37^ (Supplementary Figure 1d). Mutation of both bromodomains (BRD2^ΔBD^) blocked BRD2 recruitment to DSBs (Fig. 1e). Further, the bromodomain inhibitor JQ1, which blocks interaction with acetylated lysines^38^, also blocked recruitment of BRD2 to DSBs (Fig. 5c), without altering the production of DSBs (Supplementary Figure 2a). BRD2 is therefore localized to DSBs through binding of its tandem bromodomains to H4Ac at the DSB.

**Figure 2:**
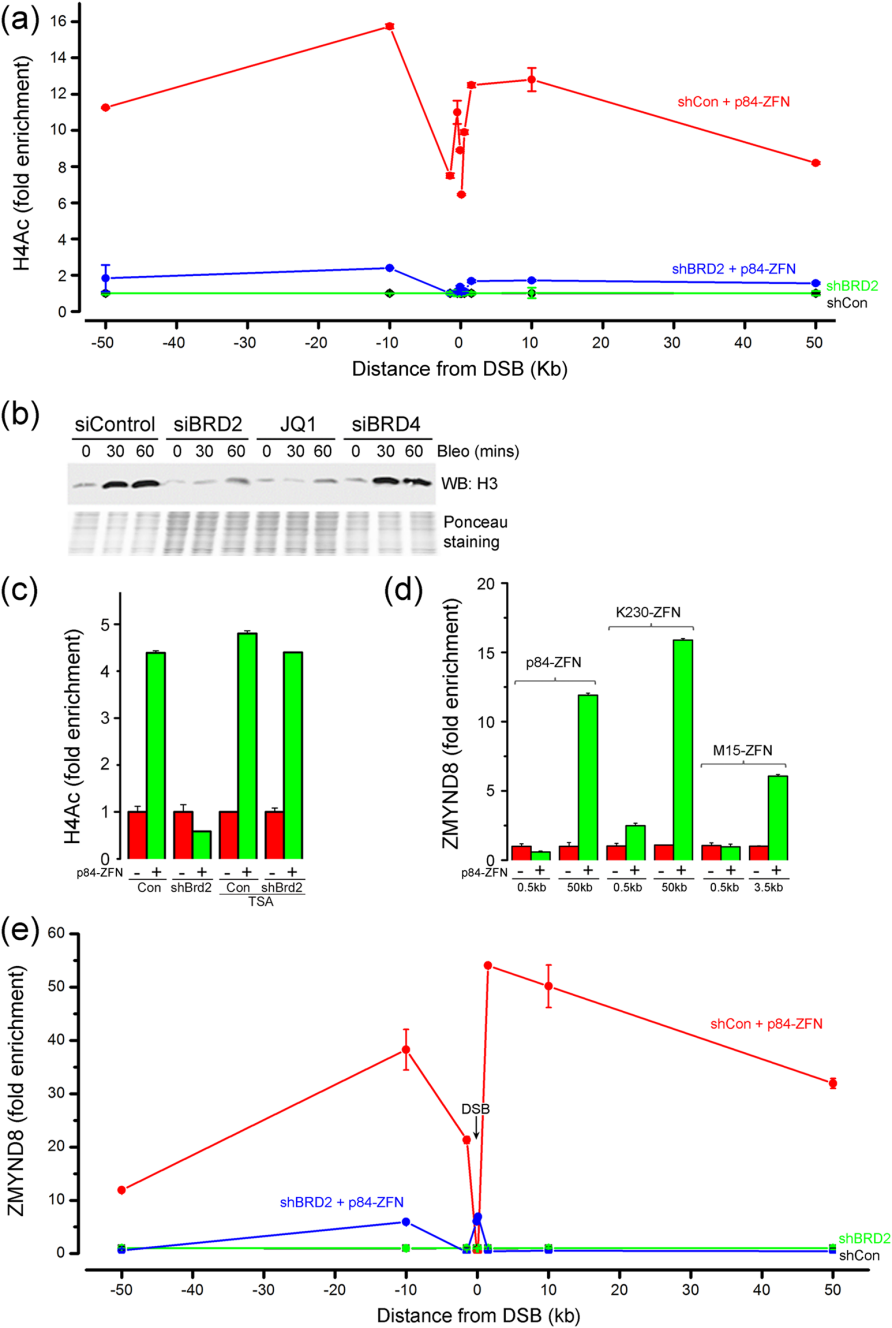
BRD2 is required for H4 acetylation at DSBs. (**a**) 293 T cells expressing control shRNA (●,) or shRNA to BRD2 (,) were transfected with vector (●,) or p84-ZFN (,), followed by ChIP with H4Ac antibody and the indicated primers. (**b**) 293 T cells were transfected with non-specific siRNA (siControl) or siRNA to BRD2 or BRD4 for 48 hrs, or incubated with JQ1 (500 nM) for 24 hrs, followed by bleomycin (7.5 μM) for 15 mins. Nuclei were prepared and extracted in 1.0 M NaCl, and histones released by NaCl extraction after DNA damage detected by western blot. Ponceau S staining confirms equal protein loading. (**c**) 293 T cells stably expressing control shRNA (Con) or shRNA to BRD2 were transfected with vector (−) or p84-ZFN (+) and TSA (400 nM), followed by ChIP using H4Ac antibody and primers 500 bp to the right of the DSB. (**d**) 293 T cells were transfected with vector (−) or various ZFNs (+). ChIP utilized ZMYND8 antibody and primers located either side of the DSB. (**e**) 293 T cells expressing control shRNA (●,) or shRNA to BRD2 (,) were transfected with vector (●,) or p84-ZFN (,). ChIP utilized ZMYND8 antibody and primers located either side of the DSB. All ChIP data calculated as IP/Input, and expressed as fold enrichment in signal relative to uncut DNA (n = 3 replicates, ± Standard Deviation). Original western images available in Supplementary Figure 7.

Next, we examined how BRD2 impacted H4Ac after DNA damage. DSBs generated broad H4Ac domains which spread at least 50 kb along the chromatin flanking the break (Fig. 2a). Surprisingly, when BRD2 expression was reduced with shRNA (Supplementary Figure 2b), there was a dramatic reduction in H4Ac across the entire flanking chromatin domain (Fig. 2a). This implies that BRD2 is required for H4Ac at DSBs. Because p84-ZFN makes a DSB in the transcriptionally active PPP1R12C gene^8,19^, we also determined if BRD2 was required for H4Ac at DSBs in intergenic regions. DSBs generated with K230-ZFN (which targets an intergenic region on chromosome 3) increased H4Ac, and this increase was also lost when BRD2 was silenced with siRNA (Supplementary Figure 2c). The ability of cells to acetylate histone H4 at DSBs is therefore dependent on BRD2.

H4Ac at DSBs creates open, flexible chromatin structures which favor repair^10,18^, implying that loss of BRD2 (and H4Ac) should lead to less mobile chromatin. H4Ac weakens histone-DNA interactions, leading to an increase in histone solubility when nuclei are extracted in high salt after DSB production^10^. Bleomycin, which creates DSBs, increased the NaCl solubility of histone H3 (Fig. 2b). Inhibition of BRD2 with siRNA (Supplementary Figure 2b) or with the bromodomain inhibitor JQ1^38^ eliminated this increase in H3 solubility (Fig. 2b). JQ1 can also inhibit BRD4, and loss of BRD4 increases chromatin relaxation and may insulate chromatin from DNA damage signals^31^, although BRD4 is not recruited to DSBs and does not act directly at DSBs^31^. Depletion of BRD4, in contrast to JQ1 or siRNA to BRD2, did not increase histone H3 solubility after DNA damage, indicating that BRD2 and BRD4 have distinct impacts on histone-DNA interactions after DNA damage. Recruitment of BRD2 to H4Ac at DSBs is therefore required to maintain H4Ac domains and promote open, flexible chromatin.

### BRD2 protects H4Ac from HDAC activity

Next, we explored how BRD2 functions to maintain H4Ac at DSBs. One potential mechanism is that BRD2 binding protects H4Ac from histone deacetylases (HDACs). The HDAC inhibitor TSA did not alter DSB production by p84-ZFN (Supplementary Figure 2a). However, TSA rescued H4Ac at DSBs in BRD2 depleted cells (Fig. 2c), implying that BRD2 is not required to acetylate H4, but instead binds to nascent H4Ac and shields it from indiscriminate HDAC activity. However, BRD2 recruitment is restricted to 4 kb either side of the DSB (Fig. 1a–c), whereas H4Ac extends at least 50 kb (Fig. 2a), suggesting that additional proteins protect the H4Ac on the flanking chromatin. One potential candidate is ZYMND8, a multivalent histone reader which binds H4Ac^29^ and H3K14Ac/H3K4me1^39–42^, and is recruited to DSBs^29,30,39^. ChIP analysis confirmed that ZMYND8 was recruited to DSBs created by either p84-ZFN, K230-ZFN or M15-ZFN (Fig. 2d). Importantly, ZMYND8 was largely excluded from the chromatin directly at the DSB (<0.5 kb: Fig. 2d), but accumulated to high levels on the flanking chromatin domains (50 kb: Fig. 2d). This is shown in more detail in Fig. 2e. ZMYND8 formed chromatin domains which spread at least 50 kb from the p84-ZFN DSB, but was largely absent from the chromatin directly adjacent to the DSB (Fig. 2d and e). BRD2 and ZMYND8 therefore occupy overlapping, but distinct chromatin domains, with BRD2 at the DSB (Fig. 1a) and ZYMND8 on the flanking chromatin (Fig. 2e). Further, when BRD2 was depleted, ZMYND8 accumulation was lost (Fig. 2e), indicating that either BRD2, or the underlying H4Ac, is required for ZMYND8 loading. Treatment of BRD2 deficient cells with TSA (which rescues H4Ac - Fig. 2c) also rescued ZMYND8 loading on the flanking chromatin (Supplementary Figure 2e), consistent with reports that ZMYND8 requires H4Ac for retention at DSBs^29^. Finally, in the absence of BRD2, ZMYND8 was detected directly at the DSB (Fig. 2e), suggesting that BRD2 may limit ZMYND8 spreading into the DSB region. However, when ZMYND8 was depleted (Supplementary Figure 2d), BRD2 accumulation at the DSB was reduced (Supplementary Figure 2f), but BRD2 did not spread into the flanking chromatin. This implies that acetylation of H4 promotes loading of BRD2. BRD2 then protects the nascent H4Ac, promoting spreading of H4Ac away from the DSB and allowing accumulation of ZMYND8 on the flanking chromatin. ZMYND8 and BRD2 therefore work together to protect H4Ac domains from HDAC activity.

### BRD2 is required to recruit 53BP1 to DSBs

**Figure 3:**
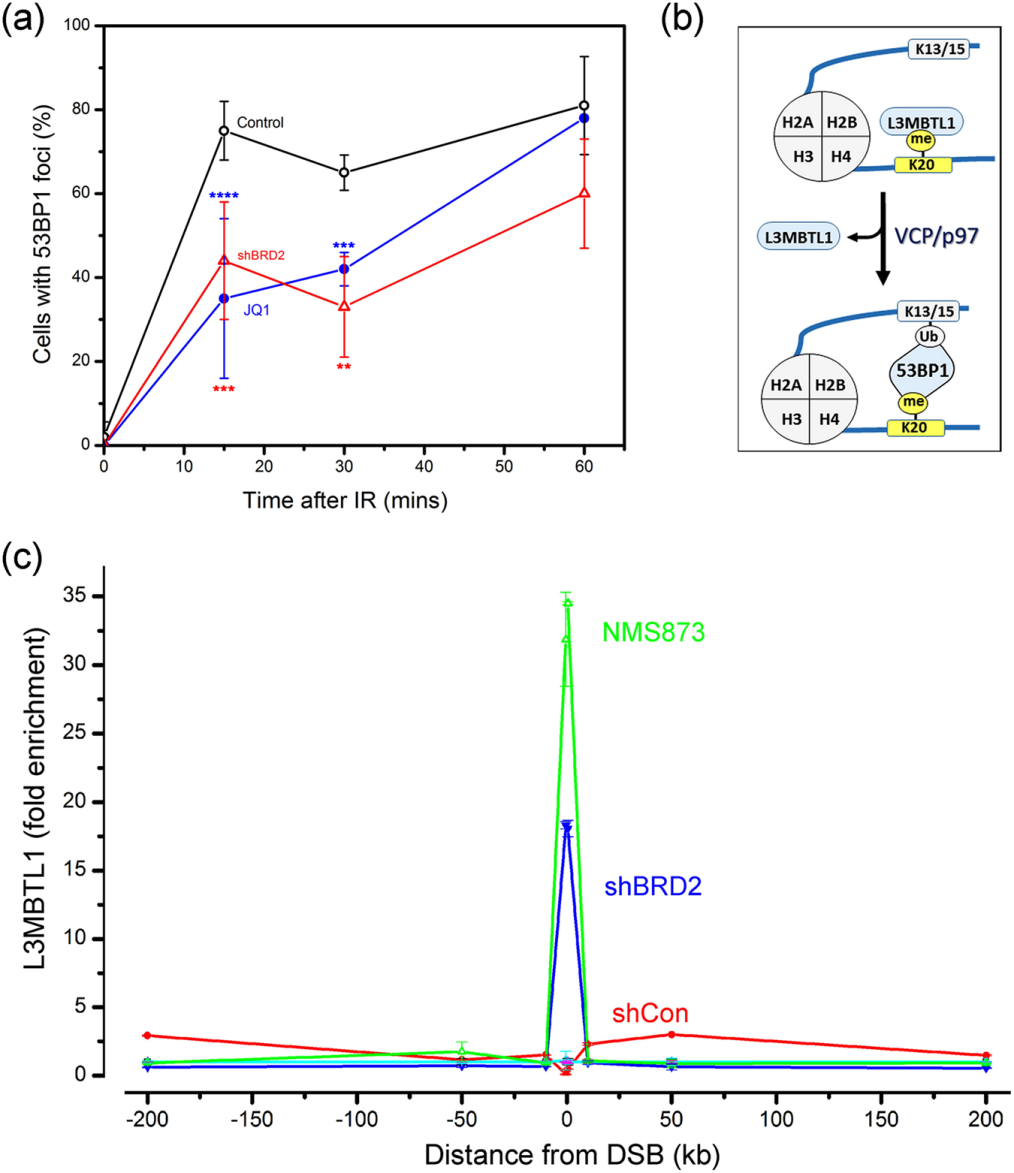
BRD2 regulates 53BP1 at DSBs. (**a**) U2OS cells were transfected with control siRNA (o), siRNA to BRD2 (), or incubated with JQ1 (; 500 nM) prior to irradiation (10 Gy). Cells were analyzed by immunofluorescent staining with 53BP1 antibody. Cells with >10 foci were counted, with 100 cells analyzed. Results ± standard deviation. *p values* calculated by ANOVA, with: **p < 0.01; ***p < 0.001; ****p < 0.0001. (**b**) VCP/p97 complex removes L3MBTL1 from H4K20me2 during DSB repair. (**c**) 293 T cells expressing control shRNA (shCon), BRD2 shRNA or treated with NMS873 (10 nM) were transfected with p84-ZFN. ChIP utilized L3MBTL1 antibody and primers located either side of the DSB.

The discovery that BRD2 binds to H4Ac at DSBs suggested that BRD2 may regulate other proteins, such as 53BP1, which also bind to the H4 tail. When BRD2 recruitment was blocked with shRNA or JQ1 (Fig. 3a and Supplementary Figure 3c) or a panel of BRD2 siRNAs (Supplementary Figure 3a), the recruitment of 53BP1 to DSBs was significantly delayed. 53BP1 recruitment can be altered during the cell cycle, but neither JQ1 nor BRD2 depletion significantly altered cell cycle position (Supplementary Figure 3b). 53BP1 recruitment to DSBs requires dual interaction with ubiquitinated H2A/H2AX^22^ and H4K20me2^24^ (Fig. 3b). However, neither DNA damage-induced chromatin ubiquitination (Supplementary Figure 3d) nor H4K20me2 (supplement Fig. 3e) were altered when BRD2 was inhibited. BRD2 depletion does not, therefore, block 53BP1 recruitment by altering either chromatin ubiquitination or H4K20me2.

### BRD2 is required for L3MBTL1 removal by VCP/p97

L3MBTL1, a tumor suppressor with high affinity for H4K20me2, can block 53BP1 binding at DSBs^25,43^. In response to DNA damage, VCP/p97, an ATPase which unfolds proteins, removes L3MBTL1 from H4K20me2, creating free H4K20me2 for 53BP1 to bind to (Fig. 3b). We used NMS872, a specific inhibitor of VCP/p97’s ATPase activity^44^, to confirm that inhibition of VCP/p97’s ATPase blocked 53BP1 recruitment but not γH2AX at DSBs (Supplementary Figure 4a) and that L3MBTL1 was lost from the chromatin at the DSB (Supplementary Figure 4b), consistent with published work^25^.

**Figure 4:**
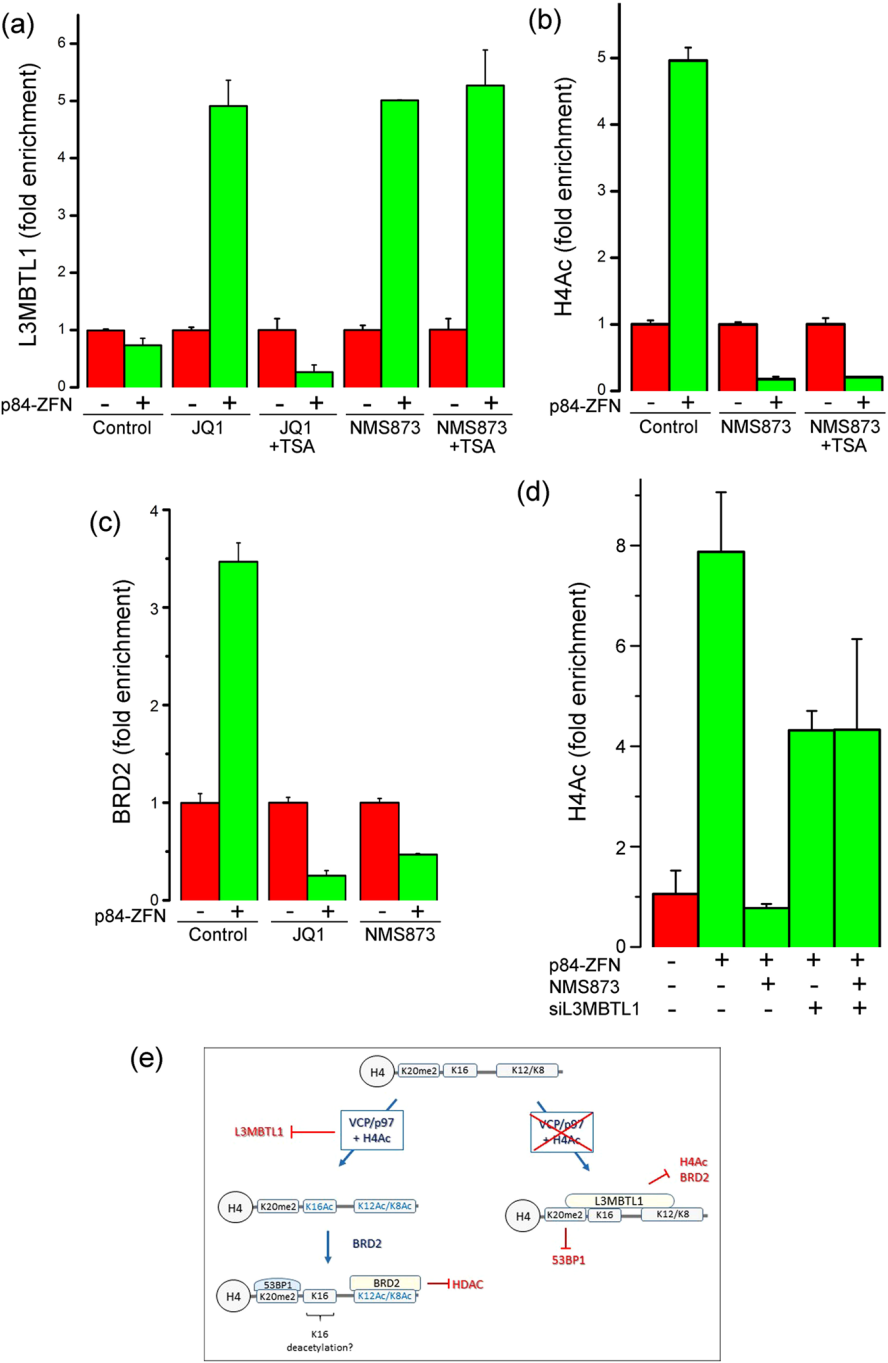
H4Ac blocks L3MBTL1 at DSBs. (**a**) 293 T cells were transfected with vector (−) or p84-ZFN (+) in the presence of DMSO (Control), JQ1 (500 nM), NMS873 (10 nM) or TSA (400 nM). ChIP utilized L3MBTL1 antibody and primers located 500 bp to the right of the DSB. (**b**) 293 T cells were transfected with vector (−) or p84-ZFN (+) in the presence of DMSO (Control), NMS-873 (10 nM) or TSA (400 nM). ChIP utilized H4Ac antibody and primers located 500 bp to the right of the DSB. (**c**) 293 T cells were incubated with JQ1 (500 nM) or NMS873 (10 nM) and transfected with vector (−) or p84-ZFN (+). ChIP utilized BRD2 antibody and primers located 500 bp to the right of the DSB. (**d**) 293 T cells were pretreated with control (−) or L3MBTL1 siRNA (+) followed by transfection with vector (−) or p84-ZFN (+). Cells were incubated with solvent (DMSO: -) or NMS873 (+) as indicated. ChIP utilized H4Ac antibody and primers located 500 bp to the left of the DSB. All ChIP data (**a**–**d**) ± standard deviation (n = 3). (**e**) BRD2/H4Ac and VCP/p97 function together to remove L3MBTL1 from chromatin at DSBs.

Next, we examined if BRD2 played a role in the removal of L3MBTL1 from H4K20me2 at DSBs. L3MBTL1 showed a complex response to DSB production. Detailed ChIP analysis revealed that L3MBTL1 was decreased at the DSB (within 4 kb), with low level accumulation of L3MBTL1 at several sites on the flanking chromatin (Fig. 3c and Supplementary Figure 4b). Unexpectedly, depletion of BRD2 did not just block L3MBTL1 removal; it led to a dramatic hyper-accumulation of L3MBTL1 which was restricted to the DSB domain (Fig. 3c). Further, inhibition of VCP/p97 with NMS873 (Fig. 4a) or depletion of VCP/p97 with siRNA (Supplementary Figure 4c,d), also led to hyper-accumulation of L3MBTL1 at DSBs. Both BRD2 and VCP/p97 are therefore required to remove L3MBTL1 from the chromatin during DSB repair and loss of either leads to dramatic accumulation of L3MBTL1. Further, both the accumulation of BRD2 at DSBs (Fig. 1a–c) and the hyper-accumulation of L3MBTL1 in the absence of BRD2 (Fig. 4c: shBRD2) were largely restricted to the chromatin domain surrounding the DSB (Fig. 4c). These results provide further evidence that the chromatin surrounding the DSB has distinct functional properties compared to the flanking chromatin, and implies that both BRD2 and VCP/p97 are required to oppose L3MBTL1 binding.

Next, we examined how BRD2 contributes to L3MBTL1 eviction at DSBs. Because loss of BRD2 leads to rapid deacetylation of H4 (Fig. 2a), we examined if both H4Ac and BRD2, or H4Ac alone, were required. JQ1 inhibits BRD2 recruitment to DSBs (Fig. 4c) and promotes accumulation of L3MBTL1 at the DSB (Fig. 4a; compare control vs JQ1). TSA, which restores H4Ac in the absence of BRD2 (Fig. 2c and Supplementary Figure 5a), prevented L3MBTL1 accumulation in JQ1 treated cells, so that L3MBTL1 was now evicted normally from the DSB (Fig. 4a; compare JQ1 vs JQ1/TSA). This indicates that it is H4Ac, rather than BRD2 bound to H4Ac, which blocks L3MBTL1 accumulation at DSBs. The contribution of VCP/p97 to L3MBTL1 eviction was then examined. Inhibition of VCP/p97 with either NMS873 or siRNA inhibited both H4Ac (Fig. 4b and Supplementary Figure 5b) and BRD2 accumulation at DSBs (Fig. 4c). Crucially, TSA did not rescue H4Ac in the absence of VCP/p97 activity (Fig. 4b) and, consequently, did not reverse the accumulation of L3MBTL1 resulting from VCP/p97 inhibition (Fig. 4a; compare NMS873 vs NMS873 + TSA). This implies that, in the absence of VCP/p97, L3MBTL1 accumulates on the H4 tail and blocks H4Ac by blocking access to the H4 acetylation sites. Thus, in the absence of VCP/p97, the high affinity of L3MBTL1 for H4K20me2 saturates all available H4K20me2 binding sites at the DSB, and functions as a dominant-negative inhibitor of H4Ac. To test this, we depleted L3MBTL1 from the cells with siRNA (Supplementary Figure 4e) and monitored H4Ac. Importantly, depletion of L3MBTL1 in the presence of the VCP/p97 inhibitor rescued H4Ac after DNA damage (Fig. 4d). L3MBTL1 binding to H4K20me2 can therefore directly block H4Ac and functions as a repressor of H4Ac by Tip60 during DSB repair.

### L3MBTL1 binding is a dominant repressor of H4Ac and 53BP1 binding

This suggests a model (Fig. 4e) in which VCP/p97 and H4 acetylation work together to prevent L3MBTL1^25,45^ from occupying the unacetylated H4 tail during DSB repair. This favors the recruitment of BRD2, which binds to H4K8Ac/K12Ac^35,36^, creating H4Ac/BRD2 domains which resist HDAC attack and further limit L3MBTL1 binding to H4K20me2. In the absence of H4Ac/BRD2 (or VCP/p97) L3MBTL1 binds to H4K20me2^25,45^ on the unacetylated H4 tail (Fig. 4e), blocking H4 acetylation by Tip60, the recruitment of BRD2 and the subsequent binding of 53BP1.

**Figure 5:**
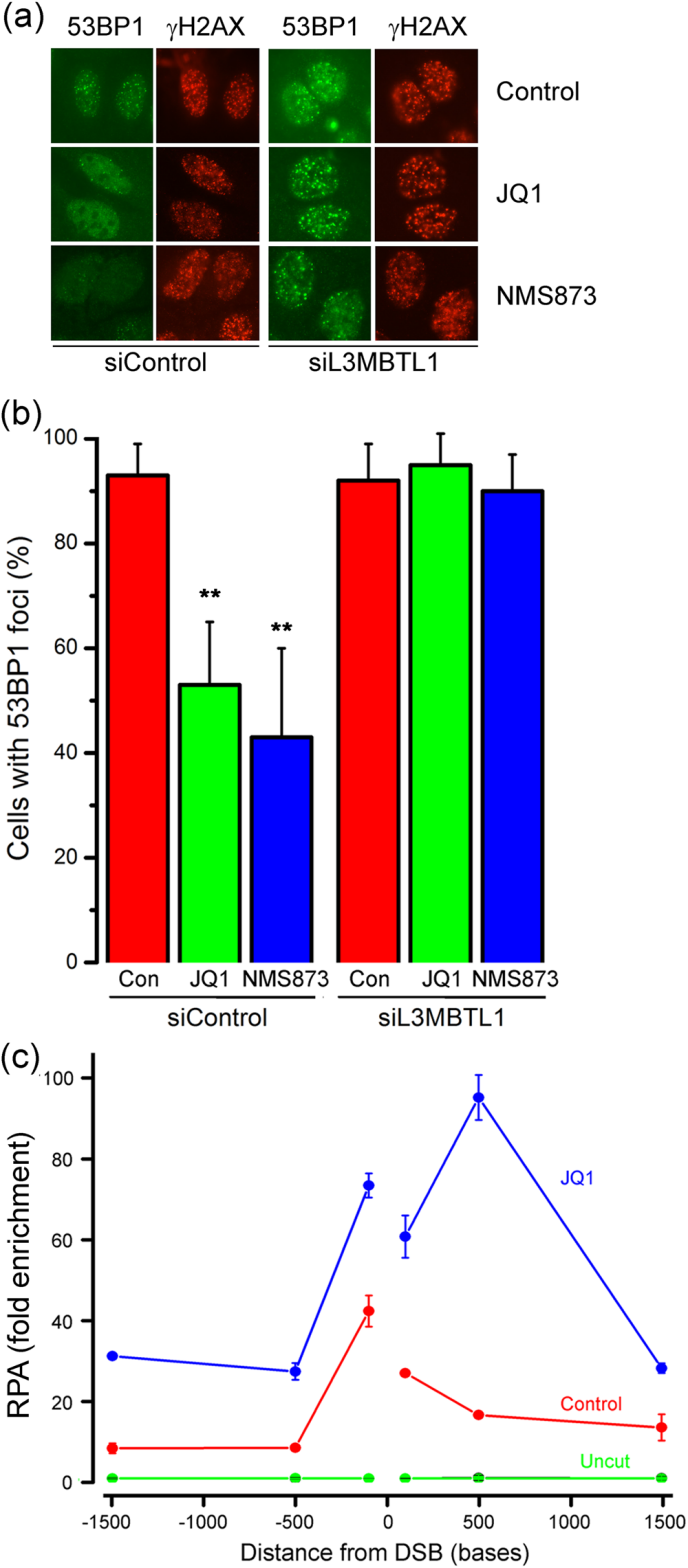
BRD2 promotes 53BP1 loading. (**a**) U2OS cells were incubated with DMSO (Con), JQ1 (500 nM) or NMS-873 (10 nM) and irradiated (10 Gy). 15 mins later, cells were analyzed by immunofluorescent staining with 53BP1 antibody. (**b**) Quantification of results in (**a**). Cells with >10 foci were counted as positive, with at least 100 cells counted per time point. Results ± SD). *p values* calculated by ANOVA, with: **p < 0.01. (**c**) 293 T cells stably transfected with vector (, ●), p84-ZFN (,) or treated with JQ1 (; 500 nM). ChIP utilized RPA32 antibody and primer pairs at the indicated positions. All ChIP data ± standard deviation (n = 3).

To test this model, we examined if depletion of L3MBTL1 could restore binding of 53BP1 in BRD2 or VCP/p97 depleted cells. Blocking either BRD2 recruitment (with JQ1) or VCP/p97 activity (with NMS873) blocked 53BP1 loading at DSBs (Fig. 4a and Supplementary Figure 6a). Importantly, depletion of L3MBTL1 rescued 53BP1 recruitment in the presence of both JQ1 and NMS872 VCP/p97. Because 53BP1 can regulate end-resection of DSBs, we also examined how BRD2 influenced production of ssDNA. RPA, a ssDNA-binding RPA complex, was used to monitor end-resection. RPA was loaded onto to a region approximately 0.5 kb either side of the DSB (Fig. 5b; Control). Inhibition of BRD2 (with JQ1; Fig. 5b) increased RPA32 binding close to the DSB, coupled with increased spreading of RPA32 to the right of the break. Loss of BRD2 therefore leads to an increase in end-resection of the break, consistent with reports that 53BP1 suppresses end-resection^46,47^. Overall, these results indicate that VCP/p97 and BRD2/H4Ac function together to remove L3MBTL1 from H4K20me2 (Fig. 4e). In the absence of BRD2/H4Ac, L3MBTL1 accumulates on H4K20me2, blocking both H4Ac and 53BP1 binding and promotes increased resection of the DSB.

### BRD2 is required for DSB repair

**Figure 6:**
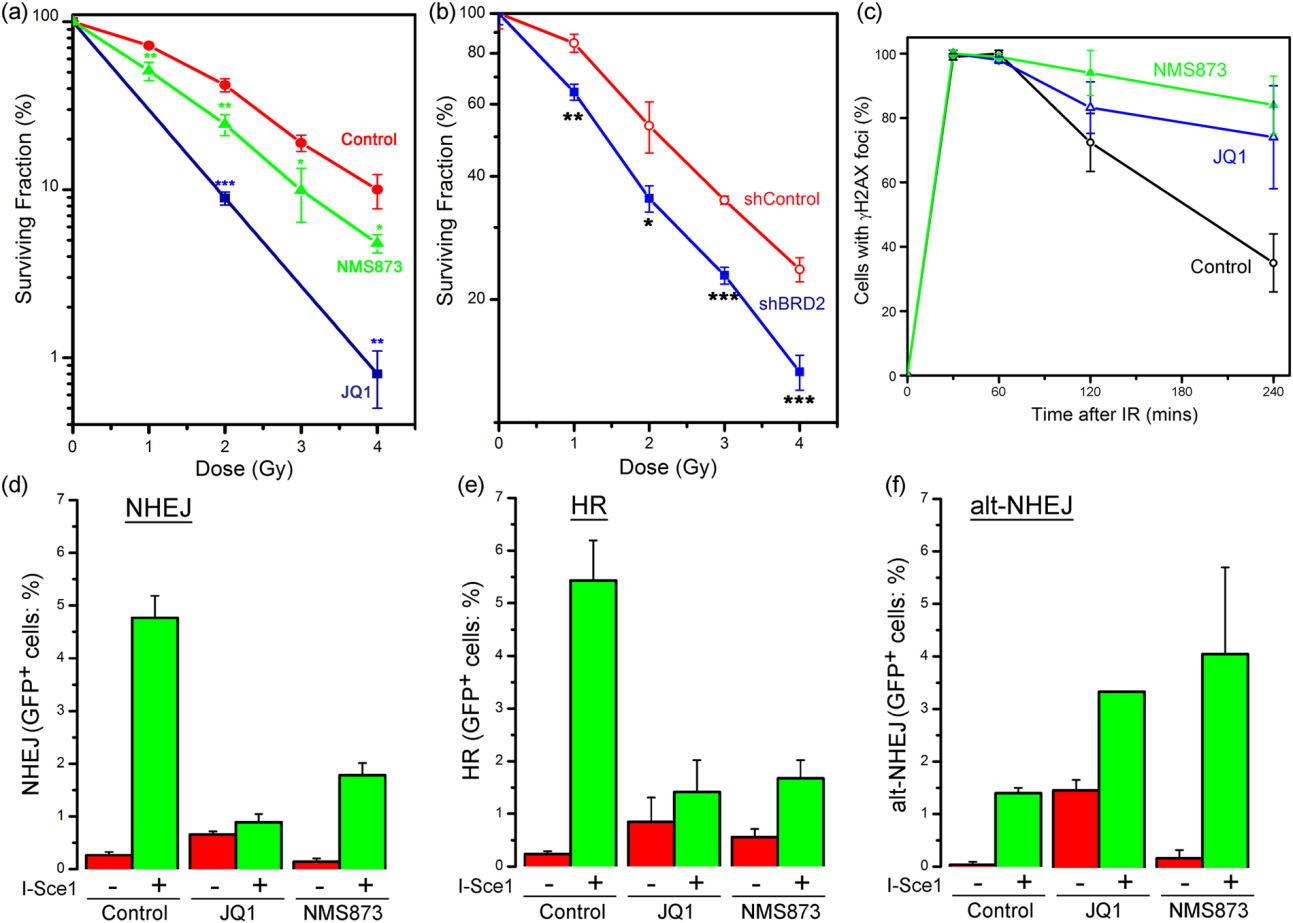
BRD2 suppresses alt-NHEJ. (**a**) 293 T cells were irradiated with JQ1 (500 nM) or NMS-873 (10 nM) and surviving colonies counted 12 days later. Resul ±SD (n = 3). *p values* calculated by ANOVA, with: *p < 0.05; **p < 0.01; ***p < 0.001. (**b**) 293 T cells stably expressing a non-specific shRNA (shControl) or BRD2 shRNA were irradiated and surviving colonies counted 12 days later. Result ± SD (n = 3). *p values* calculated by ANOVA, with: *p < 0.05; **p < 0.01; ***p < 0.001. (**c**) U2OS cells were incubated with DMSO (Control: o), JQ1 ( - 500 nM) or NMS-873 ( - 10 nM) prior to irradiation (10 Gy). Cells were analyzed by immunofluorescent staining with antibodies to γH2AX. Cells with >10 foci were counted, with at least 100 cells analyzed. (**d**–**f**) Cells with integrated NHEJ reporter (**c**), HR-GFP reporter (**d**) or alt-NHEJ reporter (**e**) were incubated in JQ1 (500 nM) or NMS-873 (10 nM), followed by transfection with I-Sce1 to create DSBs. 48hr later GFP positive cells were measured by FACs. Results ± standard deviation, with N = 3 biological replicates.

Both JQ1 and NMS873 (Fig. 6a) and shRNA to BRD2 (Fig. 6b) significantly increased radiosensitivity, consistent with a key role for BRD2 in DSB repair. JQ1 can target several BET proteins, including BRD2 and BRD4^38^, which may contribute to the higher radiosensitization by JQ1 compared to NMS873 or shRNA to BRD2. Cells treated with JQ1 or NMS872 also retained large numbers of γH2AX foci post-irradiation (Fig. 6c), consistent with cells lacking BRD2 exhibiting a defect in DSB repair. Further, cells treated with JQ1 or NMS873 had reduced repair by the NHEJ (Fig. 6d) and HR (Fig. 6e) pathways, but an increase in repair by the alt-NHEJ pathway (Fig. 6f). Interestingly, JQ1 alone appeared to increase basal activity by the alt-NHEJ pathway (Fig. 6f). Because JQ1 targets multiple BRD proteins^38^, the increase in basal GFP expression may reflect altered chromatin remodeling or chromatin organization following JQ1 addition (Fig. 6f). We therefore also used siRNA to silence BRD2 expression (Supplementary Figure 6). siBRD2 did not alter basal alt-NHEJ activity, indicating that JQ1 alters basal GFP expression by an indirect (non-BRD2) mechanism. However, siBRD2 still led to a significant increase in alt-NHEJ activity compared to controls (Supplementary Figure 6). The ability of JQ1 to increase basal alt-NHEJ activity may therefore reflect the ability of JQ1 to target multiple BRD proteins. Figure 6f and Supplementary Figure 6 therefore demonstrate that loss of BRD2 leads to a switch from NHEJ and HR-mediated repair towards alt-NHEJ. Previous work has also shown that 53BP1 can repress alt-NHEJ^47,48^. This is consistent with our results, since loss of BRD2 decreased 53BP1 loading (Fig. 3a) and increased alt-NHEJ (Fig. 6f). Further, although there is an increase in end-resection when BRD2 is inhibited (Fig. 5c), this is not reflected in an increase in HR. This implies that in the absence of BRD2 (and 53BP1), end-resection becomes deregulated, and is channeled into alt-NHEJ (which utilizes ssDNA homology) rather than classical HR. Further, this suggests that BRD2 and H4Ac may function to suppress alt-NHEJ, whereas L3MBTL1 (which limits 53BP1 loading) may promote alt-NHEJ.

## Discussion

BRD2 and ZMYND8 were recruited to a specialized, bipartite chromatin domain in which BRD2 occupies a spatially restricted region at the DSB while ZMYND8 spreads along the flanking chromatin. Since both BRD2 and ZMYND8 bind to acetylated histones^29,30,32,34,36,39^, this raises the question of how this spatial separation is maintained. ZMYND8 is a multivalent histone reader containing linked PHD-BRD-PWWP domains which recognize both H4Ac and H3K14Ac/H3K4me1^39,40^, whereas BRD2’s tandem bromodomains bind H4K8Ac/K12Ac^34–36^. Because BRD2 and ZMYND8 recognize different histone modifications, spatial separation of ZMYND8 and BRD2 may be dictated by differences in histone modifications at the DSB and on the flanking chromatin. Many histone modifications are unevenly distributed at DSBs. For example, γH2AX and H4Ac spread for 100s of kb along the flanking chromatin^10^, while H3K9me3^4^ and H2A.Z exchange during repair^8,9,49^, are restricted to within <5 kb of the DSB. Interestingly, BRD2 preferentially associates with nucleosomes containing H2A.Z^37^, suggesting that the high density of H2A.Z-nucleosomes at the DSB may favor BRD2 over ZMYND8. In addition, ZMYND8 co-recruits the NuRD-HDAC complex to DSBs^29,30^, which may selectively alter the acetylation signature on the flanking chromatin to favor ZMYND8 binding over BRD2. Further, recent work has indicated that ZMYND8 recruitment to DSBs requires the H3K4 demethylase KDM5A^41,42^. KDM5A specifically demethylates local H3K4me3 at DSBs and this demethylation is required to allow stable association of ZMYND8 with the chromatin. Specificity for BRD2 over ZMYND8 at the DSB may therefore depend on differences in histone variants and the underlying epigenetic signature at the DSB compared to the flanking chromatin. Further, it indicates that extensive editing of the local histone epigenetic signature is a key part of the chromatin response to DNA damage^41^.

The functional importance of recruiting distinct bromodomain proteins to the DSB and the flanking chromatin is unclear. BRD2 promotes transcription and increased nucleosome mobility^34^, whereas ZMYND8 is a repressive factor which limits transcription during DSB repair^29^. BRD2 may increase local nucleosome mobility at the DSB and promote DSB repair, while loading ZMYND8 on the flanking chromatin may limit large scale motion and suppress transcription during ongoing repair. Unravelling the underlying epigenetic signatures which specify BRD2 and ZMYND8 binding at DSBs will provide insight into these processes.

A key function for recruitment of BRD2 to nascent H4Ac was to protect the underlying H4Ac from attack by HDACs. Hyperacetylated chromatin domains are required for DSB repair^1,18^, and loss of H4Ac is associated with severe repair defects. The ability of BRD2 (and ZMYND8) to insulate H4Ac domains from HDACs, including HDAC complexes recruited to DSBs^29,50^, indicates the importance of maintaining H4Ac during repair. Intriguingly, BRD2 is required for spreading of H4Ac away from the DSB and loading of ZMYND8, indicating that BRD2 is upstream of ZMYND8. How localization of BRD2 to the DSB promotes spreading of H4Ac and ZMYND8 far beyond the restricted BRD2 domain is unclear. Previous work has shown that several DNA damage induced modifications, including γH2AX^51^ and H3K9me3^4^, are initiated at the DSB and then spread linearly away from the site of damage. BRD2 may bind to nascent H4Ac at the DSB, protecting it from HDAC activity and facilitate spreading of H4Ac along the damaged chromatin. In this case, BRD2 may promote spreading of H4Ac by simply binding to and shielding H4Ac from HDACs. In addition, BRD2 may also alter the local nucleosome organization at the DSB to promote access to the H4 tail by the Tip60 acetyltransferase. In the absence of BRD2, local H4Ac is reduced (and exposed to HDAC activity), and H4Ac fails to spread, preventing both BRD2 and ZMYND8 loading. However, given the complexity of ZMYND8’s histone reader activity, which includes both histone acetylation and methylation binding domains^30,39,40,42^, the loss of ZMYND8 binding and H4Ac in the absence of BRD2 may not simply reflect the loss of H4Ac spreading from the break. As an alternative, BRD2 may promote editing of other histone modifications at the DSB which may influence ZMYND8 binding. For example, removal of H3K4me3 may be required for binding of ZMYND8 to the damaged chromatin^42^. If H3K4 demethylation also requires either BRD2 or H4Ac, then loss of BRD2 would prevent H3K4me3 demethylation and loading of ZMYND8. This would leave any nascent H4Ac on the flanking chromatin exposed to HDACs and therefore lead to loss of both ZMYND8 and H4Ac on the flanking domains. A more detailed analysis of histone methylation and acetylation signatures surrounding DSBs is needed to address this issue.

Previous studies have shown that VCP/p97 plays a central role in DSB repair by preventing accumulation of L3MBTL1 at DSBs^25,52^. Our results now demonstrate that both BRD2/H4Ac and VCP/p97^25^ work together to prevent L3MBTL1 from accumulating on H4K20me2 at DSB. This co-operation is largely mediated by H4Ac, since loss of BRD2 is rescued by HDAC inhibition. We propose that acetylation of histone H4 limits L3MBTL1 binding to H4K20me2 and thereby favoring its removal by VCP/p97. This is likely to be important during the initial nucleosome processing which occurs immediately after DSB production. During DSB repair, there is a transient nucleosome compaction mediated by binding of the unacetylated H4 tail to the acidic patch on adjacent nucleosomes^8,11,12^, followed by nucleosome reorganization and release of the unacetylated, H4K20me2 tail from the nucleosome surface (Fig. 5e)^9,16,17,49^. This is a critical moment, because L3MBTL1 has a higher affinity for H4K20me2 than 53BP1^25,45^, and may rapidly bind to all the available H4K20me2. Rapid acetylation of histone H4 (by Tip60) and VCP/p97’s ATPase activity are therefore essential to prevent L3MBTL1 from occupying H4K20me2 on the unacetylated H4 tail. This creates stable H4Ac/BRD2 domains which are resistant to HDAC attack and which block L3MBTL1 binding to the adjacent H4K20me2. In the absence of either H4Ac/BRD2 or VCP/p97 (Fig. 5e) L3MBTL1 binds constitutively to H4K20me2^25,45^, blocking both 53BP1 binding and H4Ac by Tip60. Further, because 53BP1 represses end-resection^21,46,47^, L3MBTL1 accumulation leads to unregulated end resection and defective DSB repair. BRD2/H4Ac at DSBs is therefore critical for limiting L3MBTL1 binding and promoting 53BP1 binding and DSB repair.

Because BRD2 protects H4Ac, and 53BP1 binding can be blocked by acetylation of H4K16^27^, H4Ac/BRD2 binding might be expected to limit, rather than facilitate, 53BP1 binding. However, BRD2 binds to H4K8Ac/K12Ac and does not interact with H4K16Ac^35,36^. BRD2 and 53BP1 can therefore potentially both associate with the same H4 tail. Several HDACs are known to be recruited to DSBs^3,27,30,50^ and these may target H4K16Ac for specific deacetylation, thereby favoring 53BP1 loading, while BRD2 binding to H4K8Ac/K12Ac would shield these acetylation sites from attack. BRD2 may therefore protect unique acetylation codes from HDAC activity and allow for precise control of H4K16Ac status and 53BP1 loading. Previous work also implicated a related bromodomain protein, BRD4, in the DNA damage response^31^. Although loss of BRD4 led to a genome wide relaxation of chromatin structure and an expansion of γH2AX domains after DNA damage, BRD4 is not recruited to DSBs^31^. BRD4 and BRD2 therefore have distinct chromatin locations and functions during DSB repair, indicating that BRD4 does not directly contribute to the BRD2-ZMYND8 domains on the chromatin at DSBs.

BRD2/H4Ac plays a critical role in creating a spatially restricted domain at the DSB which facilitates ejection of L3MBTL1 by VCP/p97, maintains precise H4Ac signatures and promotes loading of 53BP1. BRD2 promotes spreading of H4Ac and loading of ZMYND8 on the flanking chromatin, which protects H4Ac from indiscriminate HDAC activity and promotes open, flexible chromatin that is required for DSB repair. Further, these results indicate that L3MBTL1 is a repressor of both 53BP1 recruitment and H4Ac at sites of DNA damage, suggesting that deregulation of L3MBTL1 in tumors may promote genomic instability and compromise 53BP1 loading. Finally, we note that JQ1 and NMS873 are both effective radiosensitizers. Although JQ1 and NMS873 can increase alt-NHEJ activity, which may lead to an increase in insertions/deletions, these compounds may prove useful in clinical settings in which modulation of 53BP1 function may provide a therapeutic advantage.

## Materials and Methods

### Cell culture

U2OS, HEK293T and HeLa cells (American Type Culture Collection, VA) were maintained in Dulbecco’s Modified Eagles Medium supplemented with 10% Fetal Bovine Sera^53^. Cells were tested for mycoplasma contamination monthly, and routinely replenished after less than 20 passages in culture. For clonogenic cell survival assays, cells were plated in triplicate on 6-well dishes and allowed to attach for 24 hr. Cells were irradiated using a ^137^Cs irradiator and allowed to recover for 10–14 days. Cells were fixed, stained with 10% ethanol containing 2.5% (w/v) crystal violet and colonies with >50 cells scored visually as previously described^54^. Protocols for transfection, establishing cell lines, western blot analysis (including antibodies) and sequence of siRNA and shRNA constructs is described in the supplementary methods section.

### DSB repair reporter assays

U2OS cells expressing the DR-GFP reporter^55^, HEK293 cells expressing the alt-NHEJ reporter^48^ or HeLa cells expressing an NHEJ reporter^48^ were treated with DMSO, JQ1 or NMS-873. 24hr later, cells were transfected with I-Sce I or eGFP (control) plasmid using Lipofectamine 2000 (Invitrogen, CA). GFP positive cells were detected 48hr later using the BD LSR II cell analyzer (BD Biosciences, CA) and data analyzed with the BD Diva software package. Data were normalized to eGFP (control) transfected cells to correct for transfection efficiency.

### DSB measurement and ChIP Assays

HEK293T cells were transfected with p84-ZFN or other ZFNs and allowed to recover for 18 hr. Cells were fixed in 1% methanol-free formaldehyde for 10 min to crosslink proteins, lysed in ChIP buffer (Cell Signaling Technology, MA, USA), sonicated, and cleared by centrifugation. Part of the supernatant was digested with proteinase K (65 °C for 2 hr), the DNA isolated by spin columns and input DNA quantitated by Real Time PCR. Equivalent amounts of chromatin were incubated with primary antibody (overnight at 4 °C) followed by protein G agarose beads precoated with sperm DNA. Immune complexes were washed in low and high salt ChIP buffers (Cell Signaling Technology, MA), eluted, incubated in NaCl (65 °C for 2 hrs) and digested with proteinase K. Purified DNA was quantitated by RT-qPCR using the Step One Plus real time PCR system (Applied Biosystems, CA). PCR protocols, primer pairs and ChIP grade antibodies are listed in supplementary methods.

DSB production was monitored using standard PCR based techniques previously described by us^10^. Genomic DNA prepared for ChIP was amplified using primer pairs located either side of the DSB (supplementary methods) by real time qPCR and the percent of DSBs estimated by the change in signal resulting from cleavage of the DNA. 18s rRNA genomic DNA signal was used to ensure equal input DNA.

### Real-Time quantitative PCR and data analysis

PCR amplification (using the Step One Plus real time PCR system from Applied Biosystems, CA) utilized 95 °C for 5 mins, followed by 33 cycles of: 30 sec @ 95 °C/ 30 sec @ 60 °C/ 30 sec @ 72 °C and a final extension step of 5 mins @ 72 °C. Serial dilutions of the starting material were used to determine the linear range of PCR amplification prior to use. 18S rRNA genomic sequences were used to standardize input genomic DNA^9,56^. Standard controls included immunoprecipitation with IgG, which yielded essentially no signal. Relative fold enrichment was optimized to the input control and expressed as IP/Input DNA. The relative increase in signal after cutting by p84-ZFN, K230 or M15 was calculated as [IP^ZFN^/Input^ZFN^]/[IP^Control^/Input^Control^]. All ChIP assays were repeated at least twice (biological replicates), with individual RT-qPCR reactions carried out in duplicate (technical replicate) and the results presented ± standard deviation.

### Immunofluorescence

Cells were fixed with PBS/paraformaldehyde (4%) 0–30 minutes after irradiation. Time between initial exposure and termination by fixation was <2 minutes. Cells were permeabilized in methanol, washed in PBS and incubated in Triton X-100 (0.2%) for 5 minutes. Cells were then washed twice in PBS and blocked with fetal bovine serum (10%) for 20 minutes. Slides were incubated with primary and secondary antibody with washing between each step, mounted with Fluoromount-G (Southern Biotech, AL) and imaged with a Zeiss AxioImager Z1 microscope equipped with an Axiocam MRc Rev.3 Color Digital Camera and Plan APO 63X/1.4 oil M27 lens (magnification 63X, aperture 1.4). Acquisition software and image processing utilized the Zeiss AxioVision software package (Zeiss Imaging, NY). Cells with >5 foci were scored as positive.

### Nucleosome stability assay

Washed cells were resuspended in buffer A (20 mM Hepes pH7.9, 0.5 mM DTT, 1 mM PMSF, 1.5 mM MgCl_2_, 0.1% Triton) containing 1.0 M NaCl for 40 minutes at 4^o^C with agitation as previously described^8–10^. Cells were collected by centrifugation at 100,000 g (Beckmann Ultracentrifuge) for 20 minutes, and the supernatant retained for western blot analysis.

### Data availability

All data generated or analyzed during this study are included in this published article and the associated supplementary data files.

## Electronic supplementary material

Supplementary information
