# Supplementary material for: Spatially restricted loading of BRD2 at DNA double-strand breaks protects H4 acetylation domains and promotes DNA repair
Source: Sci Rep. 2017 Oct 10;7:12921. doi: 10.1038/s41598-017-13036-5 (PMC5635005; doi:10.1038/s41598-017-13036-5)
Supplement: Supplementary file 1 — Supplementary information [file 41598_2017_13036_MOESM1_ESM.pdf]

<u>Supplementary Figures and Methods.</u>

Spatially restricted loading of BRD2 at DNA double-strand breaks protects H4 acetylation domains and promotes DNA repair.

Ozge Gursoy-Yuzugullu, Chelsea Carman and Brendan D. Price

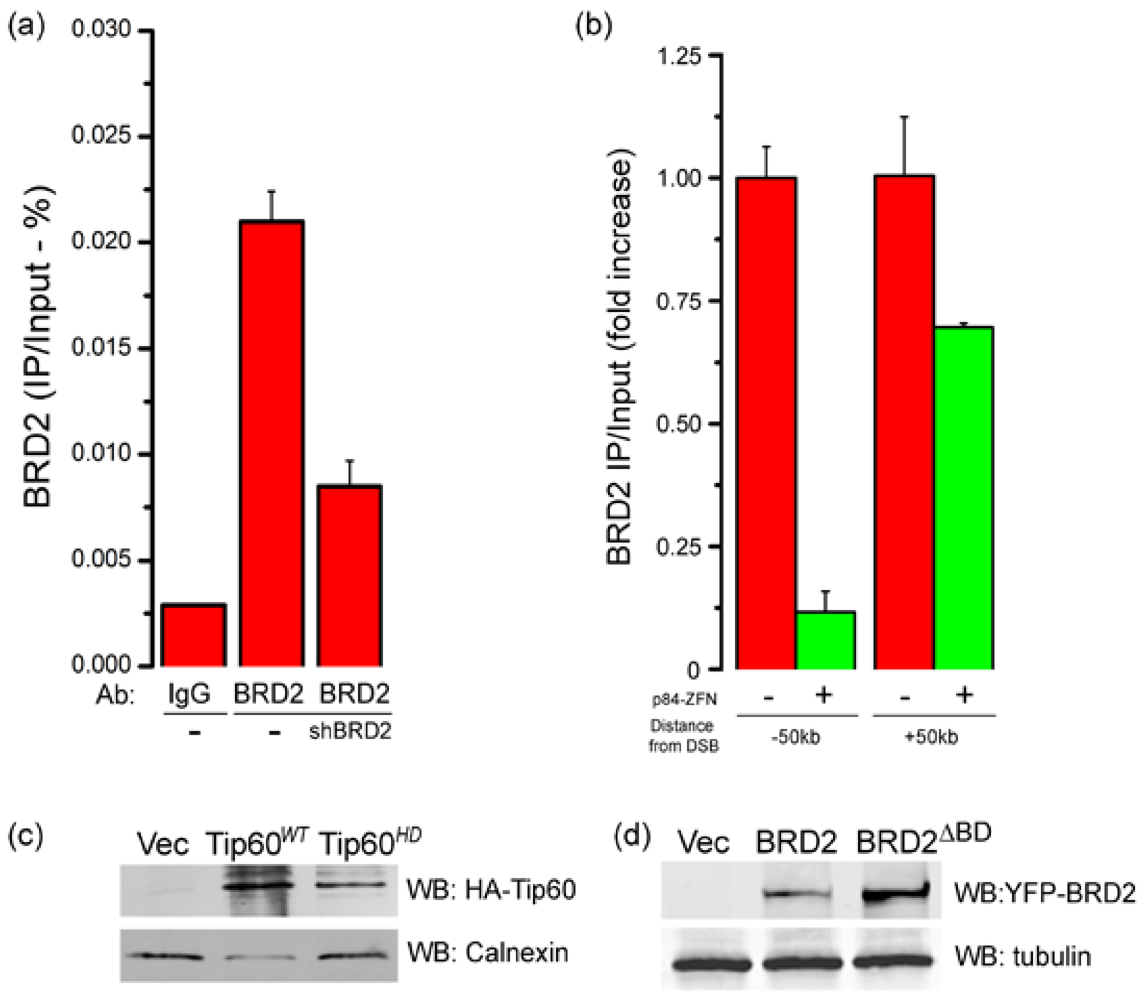

<u>Supplementary figure 1:</u>

ChIP controls, expression of Tip60 and shRNA to BRD2.

(a) 293T cells or 293T expressing control shRNA or shRNA to BRD2 were transfected with p84-ZFN (+). 18hr later, cells were processed for ChIP using either IgG or BRD2 specific antibody. Results expressed as IP/Input (%). <u>*CONCLUSION:*</u> Data demonstrates that BRD2 antibody specifically immunoprecipitates BRD2, with minimal signal when using IgG or when BRD2 levels are reduced by shRNA. **(b)** 293T cells were transfected with vector (-) or p84-ZFN (+). 18hr later, cells were processed for ChIP using BRD2 specific antibody and primer pairs located 50kb either side of the DSB. <u>*CONCLUSION*</u>: BRD2 is depleted from the chromatin 50kb upstream and downstream of DSBs created by p84-ZFN. **(c)** 293T cells stably expressing HA-Tip60^{WT} or catalytically inactive HA-Tip60^{HD} were analyzed by western blot for Tip60 expression using HA antibody, with calnexin used as a loading control. **(d)** 293T cells expressing either vector,

YFP-BRD2 or YFP-BRD2$^{\Delta BD}$, which contains a single point mutation in each of the tandem bromodomains to abolish interaction with acetylated histone H4, were analyzed by western blot for BRD2 expression using YFP antibody. Tubulin shown as loading control. Original western images available in supplementary figure 7.

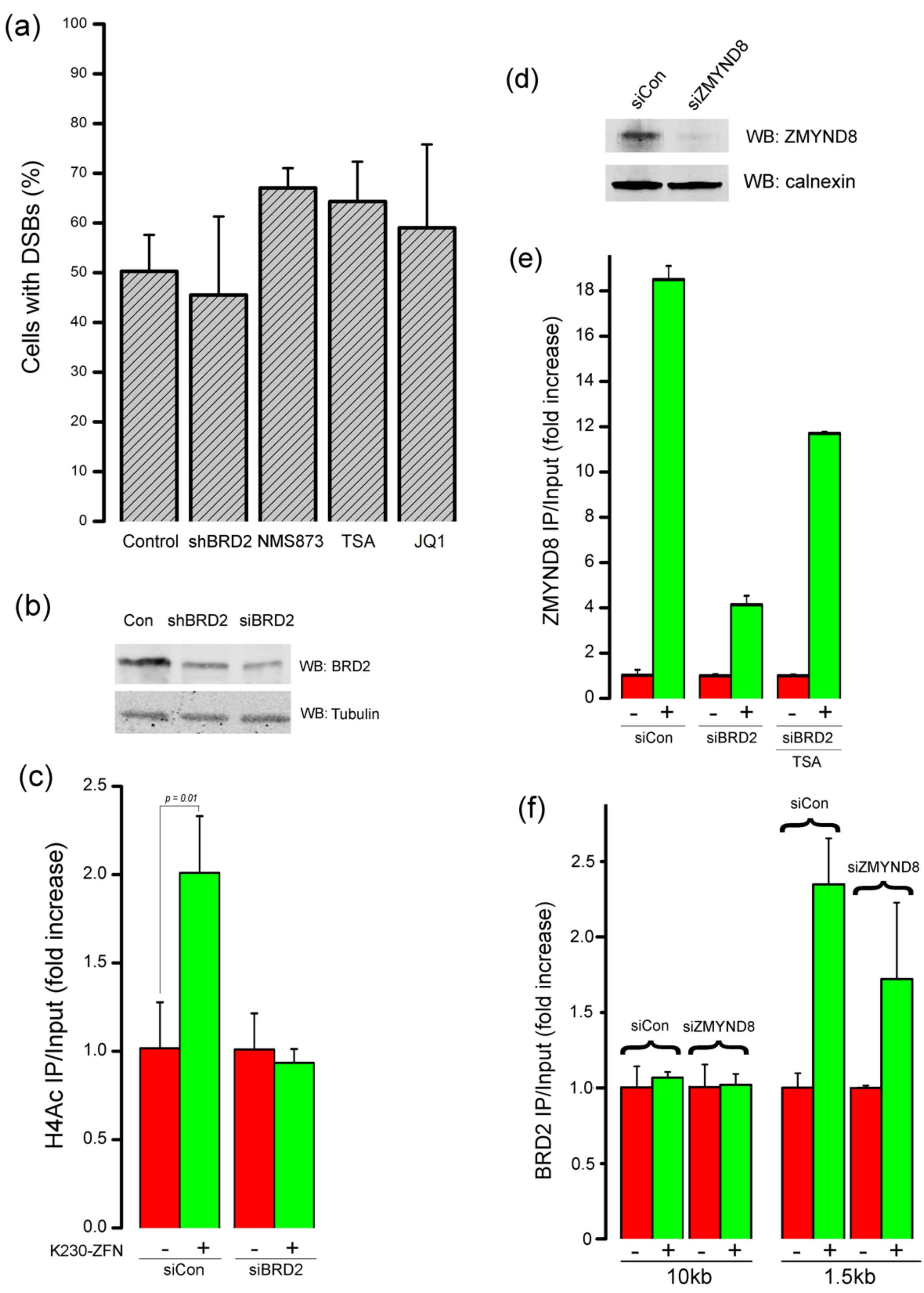
(a)
Cells with DSBs (%)
0
10
20
30
40
50
60
70
80
90
100
Control
shBRD2
NMS873
TSA
JQ1
(b)
Con
shBRD2
siBRD2
WB: BRD2
WB: Tubulin
(c)
H4Ac IP/Input (fold increase)
0.0
0.5
1.0
1.5
2.0
2.5
p = 0.01
K230-ZFN
-
+
-
+
siCon
siBRD2
(d)
siCon
siZMYND8
WB: ZMYND8
WB: calnexin
(e)
ZMYND8 IP/Input (fold increase)
0
2
4
6
8
10
12
14
16
18
-
+
-
+
-
+
siCon
siBRD2
siBRD2
TSA
(f)
BRD2 IP/Input (fold increase)
0
0.5
1.0
1.5
2.0
2.5
siCon
siZMYND8
siCon
siZMYND8
-
+
-
+
-
+
-
+
10kb
1.5kb

Supplementary figure 2:

Impact of shRNA and drug treatment on DSBs, BRD2/ZMYND8 expression and BRD2 recruitment to DSBs.

(a) 293T cells were transfected with p84-ZFN in the presence of shBRD2, or with NMS873 (10nM), TSA (400nM) or JQ1 (500nM) added immediately after transfection. 18hr later, the percent of cells with DSBs was measured by Real Time quantitative PCR using primer pairs located either side of the break. The detailed methodology is described in the methods section. *CONCLUSION*: Treatment of cells with various inhibitors does not significantly alter the efficiency of DSB production by p84-ZFN. **(b)** 293T cells stably expressing either control (shCon) or BRD2-specific shRNA, or transfected with siRNA to BRD2 were analyzed by western blot for expression of BRD2 protein. Tubulin used as loading control. **(c)** 293T cells transfected with control siRNA (siCon) or siRNA targeting BRD2 were transfected with vector (-) or K230-ZFN (+). 18hr later, cells were processed for ChIP using H4Ac specific antibody and primer pairs located 500bp to the left (-10kb) of the DSB. Results $\pm$ SD, with *p-value* calculated by ANOVA analysis. **(d)** 293T cells stably expressing either control (siCon) or ZMYND8-specific siRNA were analyzed by western blot for expression of ZMYND8 protein. Calnexin used as loading control. **(e)** 293T cells transfected with control siRNA (siCon) or siRNA targeting BRD2 were transfected with vector (-) or p84-ZFN (+) followed by TSA (400nM). 18hr later, cells were processed for ChIP using ZMYND8 specific antibody and primer pairs located 10kb to the left (-10kb) of the DSB. **(f)** 293T cells stably expressing either control siRNA (siCon) or siRNA targeting ZMYND8 (siZMYND8) were transfected with vector (-) or p84-ZFN (+). 18hr later, cells were processed for ChIP using BRD2 specific antibody and primer pairs located at the indicated distance from the DSB. *CONCLUSION*: (i) TSA can rescue loading of ZMYND8 on the flanking chromatin in the absence of BRD2, indicating that it is H4Ac which is important for ZMYND8 retention. (ii) Depletion of ZMYND8 reduces overall loading of BRD2 at the DSB, but does not completely abolish it. Further, loss of ZMYND8 does not lead to spreading of BRD2 onto the flanking chromatin domains. This indicates that ZMYND8 does not block BRD2 spreading and that restriction of BRD2 to the region adjacent to the DSB is likely defined by the underlying chromatin structure. Original western images available in supplementary figure 7.

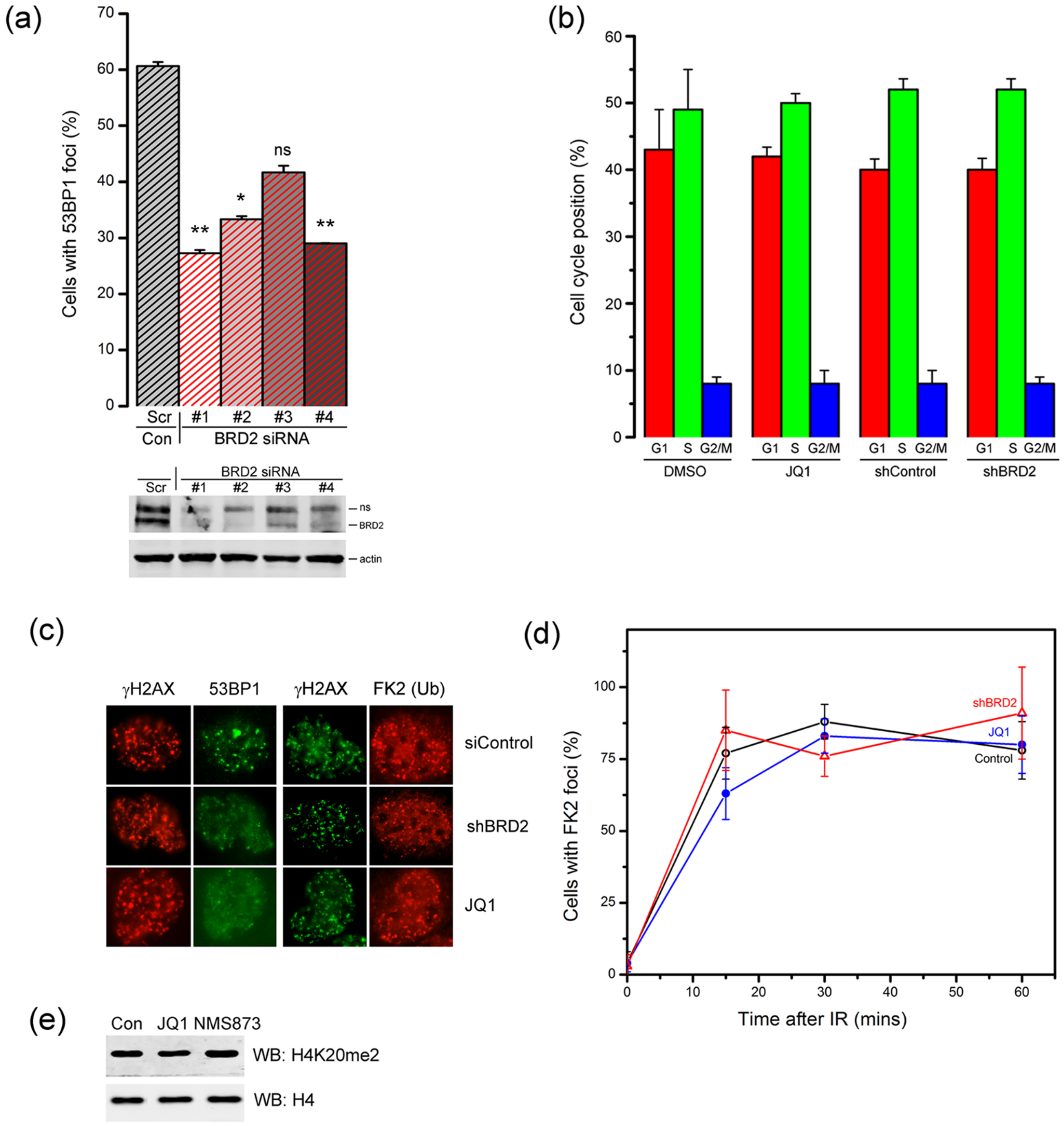

<u>**Supplementary figure 3:**</u>

JQ1 and NMS873 do not alter either H4K20me2, γH2AX or chromatin ubiquitination in response to DNA damage.

(a) U2OS cells were transfected with scrambled siRNA (scr) or 4 siRNAs targeting BRD2 - #1, #2, #3 and #4. 48hrs later, cells were irradiated (10Gy) and allowed to recover for 15 minutes. Cells with >10 53BP1 foci were counted. (Results $\pm$ SD: **, p <0.01; *, p< 0.05. ns = not statistically different from control). Western blot showing reduction in

BRD2 protein levels shown below figure. *CONCLUSION:* siRNAs 1,2 and 4 target BRD2 and can block 53BP1 loading. **(b)** Asynchronously growing U2OS cells were incubated in DMSO or JQ1 for 4hr and then analyzed by PI staining and FACS for cell cycle distribution. In addition, asynchronously growing populations of U2OS cells containing either control shRNA or shRNA to BRD2 were fixed and stained with PI and then analyzed by FACS. *CONCLUSION*: Cells exposed to JQ1 for 4hrs or in which BRD2 has been depleted do not exhibit significant differences in cell cycle kinetics compared to parental cells. **(c)** U2OS cells were transfected with control siRNA (siControl), siRNA to BRD2 or incubated with JQ1 (500nM) and irradiated (10Gy). 15mins later, cells were analyzed by immunofluorescent staining with antibodies to γH2AX, 53BP1 or the ubiquitin-specific antibody FK2. **(d)** U2OS cells were transfected with control (●) or BRD2 specific shRNA (●), or incubated with JQ1 (● – 500nM) prior to irradiation (10Gy). Cells were allowed to recover for the indicated time, fixed and analyzed by immunofluorescent staining with ubiquitin-specific FK2 antibody. No statistically significant differences in FK2 foci were found under all conditions tested ($p > 0.1$; One way ANNOVA test). *CONCLUSION:* JQ1 or loss of BRD2 do not alter the appearance of FK2 foci, indicating that loss of BRD2 does not interfere with general DNA damage-dependent ubiquitination. **(e)** 293T cells were incubated with JQ1 (500nM) or the VCP/p97 inhibitor NMS873 (10nM) and levels of histone H4K20me2 and total histone H4 measured by western blot. *CONCLUSION:* JQ1 or loss of BRD2 do not alter ubiquitination of H2AX or methylation of H4K20. Original western images available in supplementary figure 7.

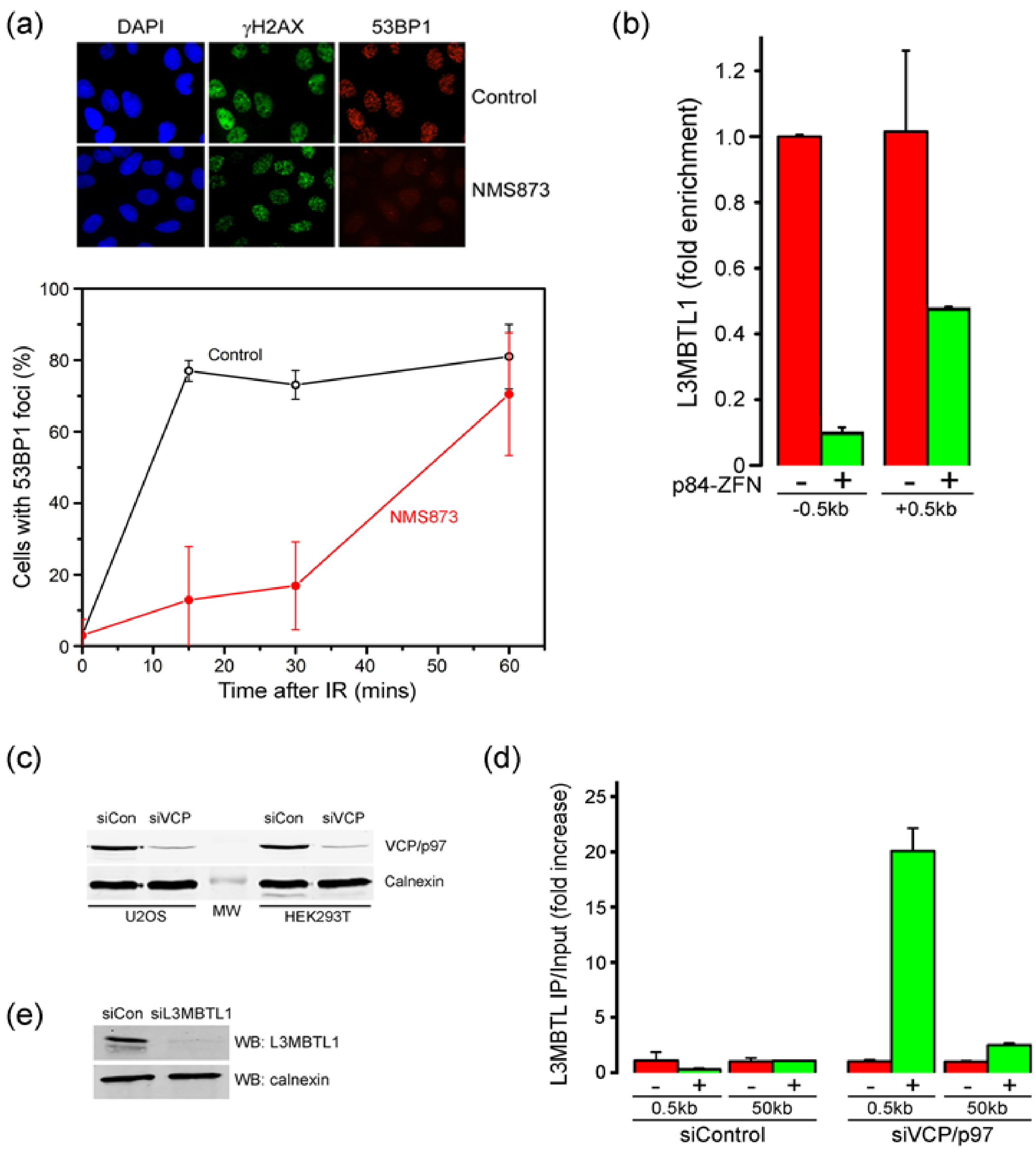

<u>Supplementary figure 4:</u>

Inhibition of VCP/p97 with NMS873 does not inhibit γH2AX formation.

(a) U2OS cells were incubated with solvent (o) or NMS873 (●; 10nM) and irradiated (10Gy). At the indicated times post-IR, cells were analyzed by immunofluorescent staining with antibodies to 53BP1. Cells with >10 foci were counted, with at least 100 cells analyzed., cells were fixed and then immunofluorescent staining carried out with antibodies to γH2AX and 53BP1. Sample images are shown. **(b)** 293T cells were transfected with p84-ZFN followed by ChIP for L3MBTL1. ChIP utilized L3MBTL1 antibody and primers located 0.5kb either side of the DSB. **(c)** HEK293T or U2OS

cells were transiently transfected with control siRNA (siCon) or siRNA targeting VCP/p97 (siVCP). 48hr post-transfection, cell extracts were analyzed by western blot for expression of VCP/p97. Calnexin shown as loading control. **(d)** 293T cells transfected with control siRNA (siCon) or siRNA targeting VCP/p97 were transfected with p84-ZFN. ChIP utilized L3MBTL1 antibody and primers located o.5kb or 50kb form the DSB. All ChIP results + SD (n = 3). **(e)** 293T cells were transfected with non-specific siRNA (siCon) or siRNA targeting L3MBTL1. Cell extracts were prepared 72hr later and examined by western blot analysis with antibody to L3MBTL1 and calnexin (loading control). Original western images available in supplementary figure 7.

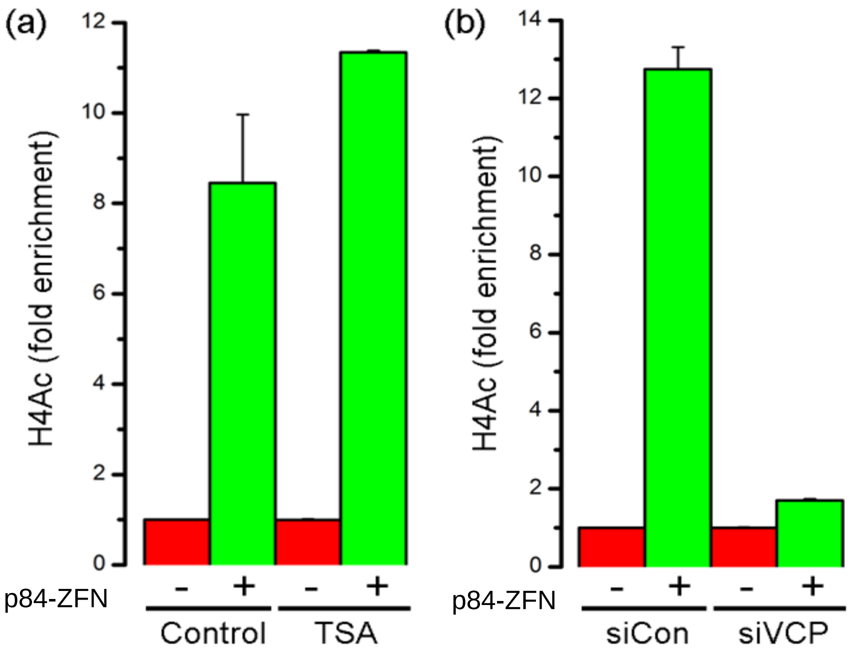

Supplementary figure 5:

VCP/p97 depletion blocks H4Ac at DSBs.

(a) 293T were incubated with DMSO (control) or TSA (400nM) followed by transfection with vector (-) or p84-ZFN (+). 18hr later, cells were processed for ChIP using H4Ac antibody and using primers located 500bp to the right of the DSB. Results $\pm$ SD (n = 3). **(b)** 293T cells were transfected with control siRNA (siCon) or siRNA targeting VCP/p97 (siVCP). 48hr later, cells were transfected with vector (-) or p84-ZFN (+). 18hr later, cells were processed for ChIP using H4Ac antibody and primers located 500bp to the right of the DSB. Results $\pm$ SD (n = 3).

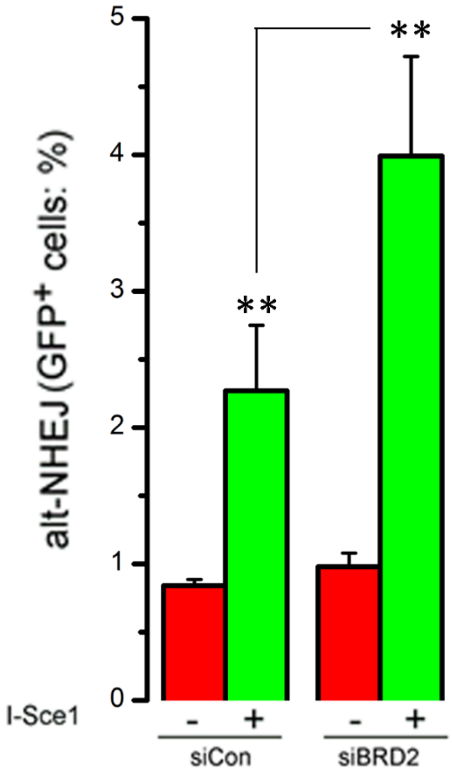

<u>Supplementary figure 6.</u>

siBRD2 increases altNHEJ.

U2OS cells with a stably integrated alt-NHEJ reporter were incubated with control siRNA (siCon) or siRNA targeting BRD2 (siBRD2). 48hr later, cells were transfected with vector or I-Sce1 and allowed to recover for a further 48hrs. GFP positive cells were then measured by FACs. Results + SD (n = 3). ** $p < 0.001$.

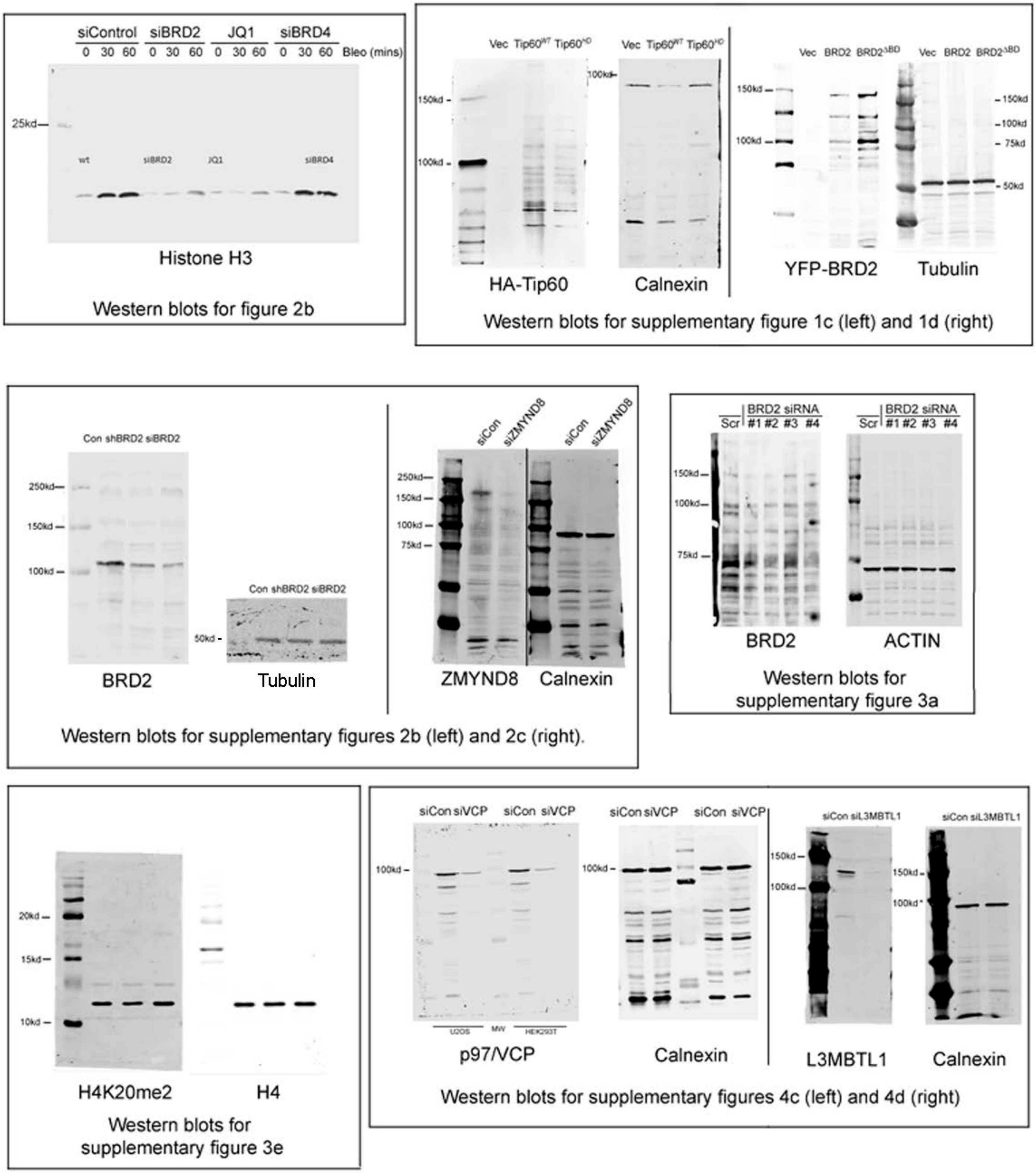

Supplementary figure 7.

Original western blot images.
